# 

**Published:** 1895-08-31

**Authors:** 


### CONTENTS.

**How Disease is Spread** - - - - - - - - - 369

**The Inspector in Excelsis** - - - - - - - - 370

**Chloroform Fatalities.—III.** By Sir W. B. Richardson - - 371

**One-and-a-Penny-Ha'penny** - - - - - - - - 372

**Medical Progress and Hospital Clinics—**

A New Classification of Ectopic Pregnancies - - - - 375

Progress in Medicine.—Rheumatism and Gout - - - 375

Progress in Surgery.—Surgery of the Spine and Cord.—Surgery of the Œsophagus and Stomach - - - - - 376

Periscope of Dermatology - - - - - - - - 380

New Appliances and Things Medical - - - - - 380

**Annotations.**—The Death of Dr. J. E. Bristowe, F.R.S., LL.D.—The Clothing of the Police—A Surgeon on Bicycles and Tricycles 373
**Notes and News** - - - - - - - - - - 374
**The Institutional Workshop—**
Within the Hospitals.—York Cottage Hospital - - - - 381
Practical Departments.—New Operating Theatre, The Middlesex Hospital - - - - - - - - - - - 382
The Story of the Insane from Year to Year - - - 383
**Construction Notes—Scraps and Gleanings** - - - - 384
**The Book World of Medicine and Science** - - - - 385
**The Student of Medicine** - - - - - - - - 385

### EXTRA SUPPLEMENT.—THE NURSING MIRROR.

**News from the Nursing World**—Our Sick and Aged Poor—Nursing Scholarships — Another Untrained Nurse — Mrs. Brown's Poultice—The Value of Testimonials—Taught to Work—Registration and Pensions—An American Training School—Receipts and Expenditure—Short Items - - - cxlvii
**Appointments** - - - - - - - - - - - - cxlix

**Elementary Physiology for Nurses.** By C. F. Marshall, M.D., B.Sc., F.R.C.S. IV.—The Circulatory System - cxlix

**The Examination in Hygiene** - - - - - - - - cxlix

**Everybody's Opinion—Notes and Queries** - - - cl

**Alumnæ Associations** - - - - - - - - - cli

**The Book World for Women and Nurses** - - - clii

